# Emerging Health Care Leaders: Lessons From a Novel Leadership and Community-Building Program

**DOI:** 10.3389/phrs.2024.1606794

**Published:** 2024-04-04

**Authors:** Andrea Martani, Agne Ulyte, Dominik Menges, Emily Reeves, Milo A. Puhan, Rolf Heusser

**Affiliations:** ^1^ Institute of Biomedical Ethics, University of Basel, Basel, Switzerland; ^2^ RAND Europe, Cambridge, United Kingdom; ^3^ Epidemiology, Biostatistics and Prevention Institute, University of Zurich, Zurich, Switzerland; ^4^ Swiss School of Public Health (SSPH+), Zürich, Switzerland

**Keywords:** public health education, public health leadership, transformative public health education, training program, transformative learning

## Abstract

**Background:**

Although there are guidelines and ideas on how to improve public health education, translating innovative approaches into actual training programs remains challenging. In this article, we provide an overview of some initiatives that tried to put this into action in different parts of the world, and present the Emerging Health Care Leader (EHCL), a novel training program developed in Switzerland.

**Policy Options and Recommendations:**

Looking at the experience of the EHCL, we propose policymakers and other interested stakeholders who wish to help reform public health education to support these initiatives not only through funding, but by valuing them through the integration of early career healthcare leaders in projects where their developing expertise can be practically applied.

**Conclusion:**

By openly sharing the experiences, strengths, weaknesses, and lessons learned with the EHCL program, we aim to foster a transparent debate on how novel training programs in public health can be organised.

## Background

The COVID-19 emergency highlighted the importance of a well-functioning public health workforce, which, in turn, requires well-designed training. Initiatives to enhance capacity building in this field already exist [1], but there is still a widespread concern that (postgraduate) training in the biomedical field is not fit for the new century [2]. In a recent contribution [3], Bürkin et al. proposed a framework how the educational approach for public health training should be changed. They recommended to break down disciplinary and professional silos, so that the public health community can come together and embrace a life-long-learning approach to education, where both academic and non-academic skills (“core human competences”) are developed. The latter are particularly important, as literature shows that organisational, managerial and leadership skills are crucial for the public health workforce of the future [4, 5].

However, translating proposals into practice requires directed efforts, since educational systems worldwide are constructed around traditional paradigms, which are not always easy to change. This is also due to the fact that innovative educational interventions are difficult to evaluate regarding their long-term impact. Therefore, reforming public health education requires also considerable courage from institutions, as they may have to commit time and resources for exploring new and unchartered territories regarding how training can be changed. It is thus key to share experiences on alternative and innovative setups of public health training, so that knowledge can circulate and (educational) institutions willing to reform their training can learn from the attempts others made in the field.

In this article, we contribute to this objective by reflecting on the Emerging Health Care Leaders project (EHCL), an innovative educational program for postgraduate early-career scientists in health service and public health research in Switzerland. First, we briefly summarise what some other comparable programs achieved in other parts of the world and then focus more specifically on the EHCL, its structure and impact. We describe the concrete policy options that have grown out of this initiative for the Swiss context, and also outline some general recommendations. Finally, we conclude by outlining the role of initiatives like the EHCL in sparking the much-needed change in public health education.

## Evidence

### Global Evidence: Leadership Initiatives for Educating the Public Health Workforce

In the last few decades, there have been several initiatives that attempted to offer educational opportunities for public health professionals reflecting some of the features proposed by Bürkin et al. and teaching non-academic skills, like leadership. In Canada, the Emerging Health Leaders is a grassroots movement that created a network of medical professionals focused on leadership development [6]. It is active since 2006 and counts hundreds of members organised in different nodes (local-committees), which organise workshops, speed-mentoring opportunities and other educational events. In Australia, the Academy for Emerging Leaders in Patient Safety yearly brings together professionals aiming to develop their leadership skills, especially for reducing the impact of medical errors [7]. This program is organised around 4-day intensive and interactive workshops, where selected participants from the professional community join “discussions, interactive presentations, storytelling, games and communication skills [training]”. It has a flat hierarchy and is focused on transdisciplinarity and inter-professionality. In the United States, upon impulses from the American Cancer Society, the National School Health Coordinator Leadership Institute was created, with the objective of developing “training for a cohort of district-level school health staff with specific goals to improve the school health infrastructure of participating school systems” [8]. This included a training program with multi-day workshops, follow-up sessions and community-building efforts over several years, addressing the development of various skills (organisational capacity, advocacy, etc.) for school health programming in participating institutions. Also in the United States, the Northeast Public Health Leadership Institute developed a comparable training opportunity, at the crossroad between academic and public health administrative institutions. Their educational offer consists in a “year-long experiential program aimed at building and improving the leadership skills of current and future public health practitioners” [9]. Their training is multidisciplinary and transprofessional and covers several topics (e.g., developing collaborative relationships, risk communication, team building, etc.). In China, a leadership training program focused around the core competences of public health and emergency preparedness identified by the American Centre for Disease Control and Prevention was developed and tested recently [10]. This included “case studies, workshops, tutorials, seminars, group discussions, role-playing, drilling, and fieldwork” (but avoided formal lecturing) on scientific, but also public communication and knowledge transfer skills.

Further initiatives of this kind are not listed here, but they have been studied and analysed in systematic reviews (e.g., [11]), but “reports on comprehensive healthcare leadership training programs (including interdisciplinary programs) are still scarce, no doubt a reflection of the paucity of such programs” [12].

### Local Evidence: The EHCL Program

In Switzerland, the training of public health professionals has traditionally focused on academic and scientific competences and on the postgraduate level. The main actors in this field include the Swiss School of Public Health (SSPH+), a partnership between 12 higher education institutions to offer PhD students from many disciplines a curriculum in public health education through workshops, conferences and dedicated resources (e.g., own certificates of advanced studies and own scientific journals) [13, 14]. Moreover, some universities developed a conjoint public health study offer [15], and a national organisation on public health offers some workshops and training opportunities[16]. Since these initiatives are mostly focused on academic skills and the development of specific projects in the field, a new initiative was launched in 2018 to complement them, offer additional (mostly non-academic) public health skill-training and enhance community building. This initiative—the EHCL program—was created in the framework of the National Research Program (NRP) 74, a funding scheme by the Swiss National Science Foundation (SNSF), which—alongside funding topic-specific projects for generating evidence around smarter healthcare for persons with chronic conditions—explicitly attempted to foster the development of a health services and public health research community [17]. The rationale was the realisation that a well-coordinated community of researchers is crucial to spark innovative and transdisciplinary research projects and to enhance the communication between public health research, practice and politics.

The EHCL was “a pioneering needs-based training programme” [18] and was open to all early career-researchers employed or involved in the research conducted as part of the 34 research-and-practice-oriented projects funded by the NRP74. Thanks to the resources of the NRP74, each eligible scholar could participate in the EHCL for free (activities were fully-funded and reimbursement for transportation to the workshops was covered), and on a voluntary basis. Over the years of its first phase, 57 early career researchers affiliated with research institution from the different language regions of Switzerland joined the EHCL. This is an important achievement given the de-centralised structure of academic and healthcare system of Switzerland. The participants were mostly PhD students (65% of the scholars), Postdocs (14%) and other researchers including junior medical doctors (21%). Many academic/professional backgrounds were present (Biology, Clinical Psychology, Nursing, Medicine, Economics, etc.). The centre of the program was a coordinating office, which initially helped with the recruitment of participants and to set up the calendar and training program, and the NRP74 steering board, a panel of experienced professionals and researchers in public health to advise and offer recommendations. A key of the EHCL was the integration of top-down approaches with a participatory/bottom-up ones, whereby scholars were increasingly involved in actively conceiving and organising new events–in terms of topic and format. This participatory approach was particularly successful. For example, the Expert Visitor Grant Program was developed, which allowed EHCL scholars to propose events involving an expert coming to Switzerland to deliver specific training in one of the educational priority-areas collectively decided.

In practical terms, the EHCL implemented a series of training workshops, events and other initiatives, which were listed in a calendar published each year. Activities included a yearly multi-day retreat, and the possibility to set up specific personalised mentorship meetings with the EHCL coordinator and experts of the Swiss healthcare community. Training topics were centred around five main thematic areas: self-competences/leadership, professional competences, soft skills, knowledge transfer competences and social competences in the working environment. With respect to the format, the events had different lengths and structures, but there were two main common foundations: the desire to comply with the principles of adult learning, and the combination—in any training event/workshop—of a skill-development and a social part for community development.

As described elsewhere [19], the EHCL managed to organise an extensive training program with a limited budget and a lean management structure. Between 2018 and 2023 more than 40 formal events were set up, on top of a series of less formalised initiatives (e.g., spontaneous discussions on/offline between scholars, *ad hoc* mentorship sessions). These were mostly skill trainings and covered a variety of topics including: leadership vs. management, team-building, successful negotiations, conflict management, personal branding, translating from evidence to policy, interactions with politics at a local and national level, presentation skills and rhetoric, writing skills in the academic and lay public domains, preparation/management of grant proposals, interactions with the media, social media for health communication, video-conferencing skills, applied project management, budgeting, etc. A full list of the events, their content, duration and name of instructors is presented in a specific detailed report [20]. Importantly, each event included a feedback-loop, whereby participating scholars could provide feedback both through short online satisfaction surveys distributed after the event, and also through the Advisory Board, a small group of selected scholars tasked to relate with the EHCL coordination office. Another key component of the EHCL was that each training event contained a social part: this consisted in shared meals before/after the event, visits to museums, short city-tours etc. This served to reach an important aim of EHCL: truly cementing the community of researchers and practitioners that were being trained. Indeed, the EHCL wanted to create a progressive increase of trust and collaboration amongst its members. This followed a step-wise approach, whereby—through the training events and the related social parts—scholars were encouraged to know and meet each other and learn about their respective skills/interests. With time, scholars were prompted to exchange ideas and start small scale collaborations, e.g., by organising some of the events for the community in cooperation with the EHCL coordinators. Finally, scholars were motivated to leverage synergies and start collaborative projects, through the availability of seed-funding. This process produced concrete and significant results, including the development of research and knowledge-transfer activities. For example, EHCL scholars worked together on a project concerning the impact of the COVID-19 pandemic on the use of primary care services [21] and also on an initiative aimed at reviewing the future trends concerning the development of the Swiss healthcare system [22]. Some other EHCL scholars initiated and then carried out a study on the influence of the COVID-19 on the use of mental healthcare in Switzerland, realised in collaboration with health insurances of the country (important stakeholders in Switzerland) [23]. Additional signs of the success of the EHCL for achieving of a sense of community-building are listed in the aforementioned report [20]. Overall, these experiences indicated that scholars expanded their professional network, developed trust and community ties and established informal connections (e.g., consulting peers about career choices). Finally, in recognition of the set of skills developed and of the new competences acquired, scholars joining a minimum number of events and skill-training workshops obtained a certificate. The certificated listed the trainings joined and the skills acquired and was signed by representatives of the NRP74 and the SNSF.

## Policy Options and Recommendations

### The Future EHCL and Lessons Learned

Finding concrete ways to improve public health education remains a challenge, in that designing innovative training programs involves a certain degree of risk and experimentation. Many countries and public health communities have started their own initiatives and it is important for policymakers who stand to interact with the future public health workforce to consider and support such initiatives. Within the context of Switzerland, the EHCL program has been trying to fill a gap in the education of health professionals. We now reflect on this experience, focusing on lessons learned that can be useful for other countries (or supranational organisation) involved in reforming public health training.

The implementation of the EHCL was initially tied to a specific NRP, which are cyclical funding and implementation programs supported by the SNSF for a specific and limited number of years. However, given the success achieved by the EHCL, several stakeholders involved in its development or implementation—as well as its scholars—acted to ensure the lessons learned would not be lost. As such, the final report of the NRP74 recommended a continuation of the EHCL, which was also communicated to different stakeholders in policy and politics [24]. This opened up the possibility to continue the EHCL program, by institutionalisation of the training offer it elaborated. This evolution is in process, through a transitional phase in which the model of training of the EHCL will continue under the umbrella of the SSPH+. An example of this new program phase was the event titled “Advocacy and Lobbying in Public Health in Switzerland: Blessing or Curse?” which was organised in collaboration with several Swiss public health stakeholders, and featured two politicians expert in public health to discuss with scholars how to interact with politics [25]. The continuation built on the lessons learned during the EHCL, including: 1) coordinators should dedicate time/support to the scholars; 2) access should be free or low-cost; 3) there should be consensus on the priority content-wise of the training; 4) evaluation of long-term effects should be planned; 5) the community of scholars should be well-connected with national and international stakeholders; 6) scholars should be open for interdisciplinary dialogue (with policymakers and non-academic stakeholders). Whereas challenges remain (e.g., setting the criteria for participation in the community), it is important for policymakers and other stakeholders in public health to continue supporting—as well as monitoring—how such innovative training initiatives function. Support of this kind does not mean only providing funding, but rather acknowledging and leveraging the value of such initiatives by interacting with them and involving early healthcare leaders in projects they can contribute to with their expertise. A key to facilitate this process is for innovative training initiatives to be transparent and communicative with other stakeholders, both concerning their strengths and weaknesses. This is the reason why the EHCL made all of its achievements, results, and details fully available, in a report written in collaboration with a science journalist to ensure accessibility [20].

## Conclusion

Public health education needs reform, and many ideas have been proposed on how this could be achieved. Whilst examples of concrete initiatives for fostering training and community-building among early-career healthcare leaders exist, they are uncoordinated and there is a risk of not learning from the experiences that many of these pioneering programs accumulated. In this article, we summarised some of such initiatives and presented the setup and some results of the EHCL program from Switzerland. We shared the content and lessons learned from this experience, so that future national and international programs can learn from the successes and the pitfalls of existing training initiatives such as the EHCL.

## Abbreviations

SNSF, Swiss National Science Foundation; NRP, National Research Program.
